# Precision Endoscopy in Peroral Myotomies for Motility Disorders of the Upper Gastrointestinal Tract: Current Insights and Prospective Avenues—A Comprehensive Review

**DOI:** 10.3390/life13112143

**Published:** 2023-10-31

**Authors:** Francesco Vito Mandarino, Edoardo Vespa, Alberto Barchi, Ernesto Fasulo, Emanuele Sinagra, Francesco Azzolini, Silvio Danese

**Affiliations:** 1 Department of Gastroenterology and Gastrointestinal Endoscopy, IRCCS San Raffaele Hospital, Vita-Salute San Raffaele University, Scientific Institute San Raffaele, 20132 Milan, Italy; 2 Gastroenterology and Endoscopy Unit, Fondazione Istituto San Raffaele Giglio, 90015 Cefalù, Italy

**Keywords:** myotomy, POEM, achalasia, gastroparesis, Zenker diverticulum, precision

## Abstract

Our review delves into the realm of peroral endoscopic myotomies (POEMs) in the upper gastrointestinal tract (UGT). In recent years, POEMs have brought about a revolution in the treatment of UGT motility disorders. Esophageal POEM, the first to be introduced, has now been validated as the primary treatment for achalasia. Subsequently developed, G-POEM displays promising results in addressing refractory gastroparesis. Over time, multiple endoscopic myotomy techniques have emerged for the treatment of Zenker’s diverticulum, including Z-POEM, POES, and hybrid approaches. Despite the well-established efficacy outcomes, new challenges arise in the realm of POEMs in the UGT. For esophageal POEM, the future scenario lies in customizing the myotomy extent to the minimum necessary, while for G-POEM, it involves identifying patients who can optimally benefit from the treatment. For ZD, it is crucial to validate an algorithm that considers various myotomy options according to the diverticulum’s size and in relation to individual patients. These challenges align with the concept of precision endoscopy, personalizing the technique for each subject. Within our text, we comprehensively examine each myotomy technique, analyzing indications, outcomes, and adverse events. Additionally, we explore the emerging challenges posed by myotomies within the context of the evolving field of precision endoscopy.

## 1. Introduction

Peroral endoscopic myotomies (POEMs) have emerged as transformative interventions for motility disorders of the upper gastrointestinal tract (UGT). These procedures follow the principles of third-space endoscopy and belong to the NOTES (Natural Orifice Transluminal Endoscopic Surgery) approach, which aims to perform surgical procedures using natural body orifices as entry points.

In the early 2000s, esophageal POEM was developed for the treatment of achalasia. This was followed by gastric POEM (G-POEM) for refractory gastroparesis (GP) and Z-POEM for Zenker’s diverticulum (ZD). More recently, new endoscopic myotomies have been providing therapeutic options for other pharyngeal-esophageal motility disorders, such as diverticula of the mid-esophagus and cricopharyngeal bar. Overall, peroral endoscopic myotomies have demonstrated optimal long-term clinical outcomes with few adverse events, leading to a shift in the management of UGT motility disorders and a significant reduction in the need for surgery. However, the evolution of these techniques presents new challenges.

Recently, the concepts of personalized medicine and precision endoscopy have been developed [1,2]. Precision endoscopy is a broad concept that involves tailoring endoscopic techniques for individual patients or diseases. This concept is also relevant to future scenarios of UGT myotomies, addressing the unique challenges posed by each procedure. It means tailoring the extent of myotomy in esophageal POEM to avoid unnecessary risks, identifying patients with refractory GP who would benefit from G-POEM, and validating a comprehensive endoscopic treatment algorithm for (ZD).

In this review, we analyze the main endoscopic myotomies for UGT motility disorders: indications, outcomes, techniques, and adverse events. Additionally, we analyze the challenge of precision endoscopy for these procedures. We propose that this approach represents the winning strategy to further enhance the performance of these techniques in improving patient outcomes.

## 2. Methods

A comprehensive literature search was conducted using PubMed, Medline, and Embase to identify all English-language articles published up to July 2023 that evaluate Peroral Endoscopic Myotomies for the management of UGT motility disorders. The considered outcomes included efficacy, endoscopic technique, and adverse events. The search was conducted using a combination of keywords, such as “Peroral Endoscopic Myotomy”, “Esophageal Peroral Endoscopic Myotomy”, “Gastric Peroral Endoscopic Myotomy”, “Zenker’s Peroral Endoscopic Myotomy”, “E-POEM”, “G-POEM”, “Z-POEM”, “Achalasia”, “Gastroparesis”, and “Zenker’s diverticulum”.

The reference lists of relevant abstracts, original articles, and reviews were reviewed, but only articles published in peer-reviewed journals were included, with a preference given to the most recent studies.

## 3. Esophageal Peroral Endoscopic Myotomy (POEM)

Over a century ago, Heller introduced a surgical intervention for esophageal achalasia, involving the dissection of the muscle fibers of the lower esophageal sphincter (LES). This procedure evolved into the modern laparoscopic Heller myotomy (LHM) with anterior fundoplication. LHM has demonstrated high efficacy and safety [3,4,5]. However, the emergence of a groundbreaking alternative in the last decade has challenged the prominence of LHM.

POEM was conceived in 2007 by Pasricha and colleagues [6] and refined by Inoue and colleagues [7]. It is a scarless procedure that creates a trans-oral submucosal tunnel in the esophagus for the direct endoscopic dissection of LES muscle fibers.

Robust data have shown that POEM offers the same efficacy, shorter recovery times, and greater patient comfort compared to surgery. The procedure has gained global recognition as a preferred treatment in several centers for achalasia. However, concerns about gastroesophageal reflux disease (GERD) have emerged as a complication due to the lack of anti-reflux procedures performed during LHM.

### 3.1. Technique

POEM is performed with the patient under general anesthesia, employing positive pressure ventilation, and using higher insufflation pressures compared to those generated by endoscopic carbon dioxide (CO_2_) insufflation. The steps described below refer to the technique described by Inoue et al. [7].

**Figure 1:**
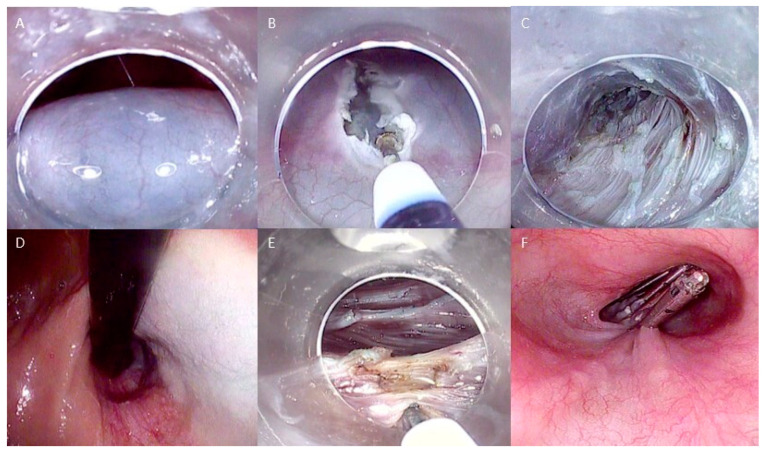
Technical steps of esophageal POEM: (**A**) Submucosal injection; (**B**) Mucosal incision to create the tunnel entrance; (**C**) Submucosal tunneling; (**D**) Control of tunnel extension in retroflexed view from the stomach; (**E**) Myotomy of the circular muscle fibers; (**F**) Closure of the mucosal incision with endoclips. The copyrights to the pictures belong to the authors.

A triangle-tip knife or a similar one, such as the T-Knife (ERBE, Tubingen, Germany), is used for all cutting steps during the intervention. Technical steps are shown in Figure 1.

The procedure begins with the creation of a mucosal entry, followed by a submucosal injection of a solution containing 10 mL of saline with epinephrine and 0.3% indigo carmine at the mid-esophageal level, approximately 10–13 cm proximal to the gastroesophageal junction (GEJ). The mucosal incision, typically longitudinal and measuring 2 cm in size, is made to access the submucosal space. A tunnel is created in a cranio-caudal direction, extending approximately 3 cm beyond the GEJ into the proximal stomach. To achieve this, the technique follows the principles of submucosal endoscopy, using the dry cut mode (50 W, effect 3) as the cutting current. The lateral extension of the submucosal tunnel is adjusted to encompass about half of the esophageal circumference. Dissection of the submucosal layer is facilitated using the spray coagulation mode (50 W, effect 2) and the soft coagulation mode (80 W, effect 5) to address larger submucosal vessels.

Sequentially, the myotomy of the circular muscle is performed, initiated approximately 3 cm distal to the mucosal entry point, situated approximately 7 centimeters above the GEJ. The triangle-tip knife is utilized to elevate and subsequently segment individual circular muscle bundles using a spray coagulation current (50 W, effect 2). The dissection continues in a distal direction towards the stomach, progressing until navigating through the narrow segment of the LES, where a noticeable transition is observed as the submucosal space expands and extends for approximately 2 centimeters into the stomach.

Upon completing the myotomy, the closure of the mucosal entry is achieved by applying approximately five hemostatic through-the-scope (TTS) clips. The procedure concludes with the reinsertion of the endoscope through the natural lumen, confirming smooth passage through the GEJ and down to the stomach.

In addition to the standard technique, various technical and procedural variations have been explored over the years. These primarily involve variations in the location of the submucosal tunnel and myotomy (anterior versus posterior wall of the esophagus) and the depth of the myotomy (full thickness versus limited inner circular muscle myotomy).

There is no global consensus regarding the anterior versus posterior approach to myotomy. In the Randomized Controlled Trial (RCT) conducted by Tan and colleagues, the comparison of the two approaches showed similar clinical efficacy, overall safety, and post-procedural manometric findings [8]. The most recent systematic review and meta-analysis of RCTs confirmed similar clinical success and GERD rates after anterior and posterior myotomies [9]. However, in the RCT conducted by Stavropoulos et al., anterior POEM was associated with more mucosal heat injuries and a slower myotomy speed and closure time [10]. It is reasonable to hypothesize that the posterior approach is safer and faster than the anterior approach due to its reduced proximity to vital structures and better visualization of the esophageal anatomy. In our center, we typically perform POEM on the posterior wall of the esophagus.

Regarding the depth of myotomy, although full-thickness myotomy was initially believed to have a major positive impact on clinical success, both limited to the inner circular layer and full-thickness have shown similar efficacy and safety [11,12,13]. The latter is preferred at our institution due to its speed.

### 3.2. Indications and Patient Selection

POEM is now an established approach for treating esophageal achalasia. While comparable efficacy rates have been shown across the three achalasia subtypes distinguished by the Chicago classification, it has been established that POEM provides distinct advantages, especially for spastic achalasia subtype 3, when compared to alternative treatments like surgery and pneumatic dilation [14]. This is due to POEMs capability to generously extend the myotomy proximally in the esophagus to ablate spastic muscular segments. Remarkably, a strategy tailored to high-resolution manometry (HRM) can enhance procedural outcomes. In the study by Kane et al., enrolling 40 patients with type III achalasia, HRM-tailored POEM was associated with a significantly greater mean myotomy length (16.6 ± 2.2 centimeters versus 13.5 ± 1.8 centimeters; *p* < 0.0001) and a more favorable post-operative Eckardt score (0.2 ± 0.4 versus 1.3 ± 1.5; *p* = 0.043) compared to the standard procedure [15].

POEM requires careful consideration for young patients with achalasia due to potential concerns. The risk of post-POEM GERD should be approached cautiously during decision-making, considering its chronicity and related complications (peptic strictures, Barrett’s esophagus, esophageal adenocarcinoma) [16]. Additionally, the long-term impact of POEM-induced esophageal anatomical changes on young individuals necessitates further understanding [17]. Despite obesity being a recognized risk factor for GERD, recent evidence did not find worse post-POEM outcomes in obese patients compared to non-obese achalasia patients [18,19].

POEM has been implemented in the therapeutic management of a wide range of esophageal spastic disorders, primarily including Esophagogastric Junction Outflow Obstruction (EGJOO), Jackhammer esophagus (JE), and Distal Esophageal Spasm (DES) [20]. In these conditions, myotomy aims to reduce the muscular tone of the distal esophagus and LES, which contribute to the onset of dysphagia [21].

### 3.3. Outcomes

#### 3.3.1. Achalasia

The Eckardt score, which ranges from 0 to 12 and includes symptoms such as dysphagia, regurgitation, and chest pain, remains the primary scoring system used for the clinical assessment of achalasia [22]. A score of ≤3 is widely accepted as indicating post-POEM clinical success [23].

POEM has demonstrated remarkable efficacy in alleviating symptoms associated with achalasia. Initial reports demonstrated clinical success exceeding 95% [7,24].

In an international retrospective multicenter study conducted by Inoue and colleagues, which included 500 achalasia patients treated with POEM, a significant decrease in the Eckardt score after the procedure persisted for 3 years of follow-up (6.0 ± 3.0 versus 1.0 ± 2.0, *p* < 0.0001) [25]. In another large single-center experience involving 400 achalasia patients, the clinical success of POEM 3 years after the procedure was 87%. The authors identified the length of the disease being over 10 years and prior treatment as the principal predictive factors for symptom recurrence [26].

Over a decade after its introduction, comprehensive long-term data has accumulated. The most recently updated meta-analysis pooled an overall clinical success rate of 82% at 5 years after POEM. Similar results were found for all achalasia subtypes (86.1%, 87.9%, and 83.9% for types I, II, and III, respectively) [27]. Zang et al. assessed the long-term efficacy of POEM with 7 years of follow-up, reporting a sustained clinical success rate of 88% [28]. In the multicenter study conducted by Modayil et al., involving 610 patients who received esophageal POEM, clinical success at years 1, 2, 3, 4, 5, 6, and 7 was 98%, 96%, 96%, 94%, 92%, 91%, and 91%, respectively [29]. Table 1 shows the main studies assessing the outcomes of POEM in achalasia patients.

#### 3.3.2. Non-Achalasia Disorders

In the first multicentric study assessing POEM in the treatment of patients with DES and JE, a clinical response rate of 93% was observed at a median of 8 months of follow-up [30]. Subsequently, a retrospective study reported lower long-term success rates (83%), along with a prevalence of post-POEM erosive esophagitis close to 50% [31]. Other series, which also included EGJOO patients, displayed success rates ranging from 80% to 87% [32,33].

A multicenter study evaluating POEM in the treatment of EGJOO, including 55 patients—of whom half had previously failed alternative interventions—reported a clinical success rate of 94% [34]. The first prospective trial on severe EGJOO treatment with POEM also demonstrated a high success rate (almost 93%) at a 6-month follow-up, accompanied by significant decreases in mean IRP and quality of life scores [35].

Non-achalasia disorders are highly heterogeneous conditions, and the decision to propose POEM must follow a meticulous diagnostic-therapeutic algorithm.

Firstly, these disorders should all present dysphagia or chest pain as clinical features, aligning them with achalasia-like disorders, thus making them candidates for myotomy. In some instances, particularly with EGJOO, symptoms may also arise from LES hypercontraction in response to acid reflux, hiatal herniation, or opioid-induced mechanisms. In these secondary variants, POEM may be highly detrimental and not indicated. For this reason, the diagnostic algorithm involves the exclusion of underlying GERD (PPI trial, pH testing), followed by diagnostic methods like Functional Luminal Imaging Probe (FLIP) or Timed Barium Esophagogram (TBE) [36].

Secondly, a meticulous, step-up therapeutic approach that includes anticholinergic, nitrate, and sildenafil pharmacological trials, followed by endoscopic dilation, is recommended for persistent symptoms. In this algorithm, POEM should only be considered in cases of inadequate response to other treatments, while keeping in mind the irreversible nature of the myotomy [21]. Under these conditions, POEM holds the potential to be an intervention with satisfactory long-term efficacy.

### 3.4. Adverse Events

POEM is a relatively safe procedure, and it should be performed in controlled settings. Intra-procedural adverse events mainly include mucosal perforations, pneumothorax, pneumoperitoneum, pneumomediastinum, subcutaneous emphysema, pleural effusion, and pneumonia. Although these events are typically self-limiting and mostly resolved during the procedure with endoscopic treatment [37], major adverse event rates are reported to vary between 1.2% and 2.2% [38,39,40].

The safety of POEM appears to be consistent even in more complex situations, such as in patients who have previously undergone surgical treatment. A recent meta-analysis by Huang et al., which assessed 9 studies involving POEM for patients with prior LHM (a total of 272 patients), reported only mild intra-procedural myotomy-related adverse events in 6 studies (mainly mucosal injury, followed by pneumothorax and pneumomediastinum) [41].

### 3.5. POEM and GERD

GERD represents the Achilles’ heel of POEM and is its most significant long-term concern. Unlike traditional surgical approaches that can include concurrent antireflux fundoplication, POEM lacks this component, potentially allowing gastric contents to flow back into the esophagus more easily.

In a prospective cohort study enrolling 58 patients who underwent POEM, Teh et al. found that 60% of subjects had reflux esophagitis at post-procedural endoscopy, with 18% classified as Los Angeles grade C and D. Concurrently, 56% of patients showed pathological reflux time exposure on pH-impedance evaluation [42].

In the study by Modayil et al., a positive pH study and reflux esophagitis were found at 4-month follow-up in 57.1% and 49.8% of the patients, respectively [29].

Identifying predictive factors for post-POEM reflux remains challenging; a comprehensive meta-analysis failed to identify statistically significant correlations between patient characteristics, achalasia sub-type, POEM orientation (anterior versus posterior myotomy), and reflux findings [18]. Conversely, a recent large international multicenter experience involving 2905 patients treated with POEM, which reported a 3-month reflux esophagitis rate of almost 65%, identified age > 65 years at the procedure, male sex, posterior orientation of myotomy, and esophageal and gastric-side myotomy lengths > 10 and > 3 cm as predictive factors for mild GERD occurrence after POEM. Interestingly, in the subgroup multivariate analysis for severe stages of endoscopic esophagitis (Los Angeles scores C and D), esophageal-side myotomy length > 10 cm (*p* < 0.001), along with age (*p* < 0.001), sigmoid-type esophagus (*p* < 0.03), previous treatments (*p* < 0.001), and an Eckardt preoperative score > 7 (*p* < 0.009), were found to be significant [43]. In a recent systematic review and meta-analysis of 11 studies involving 2342 patients, long-term (>2 years) reflux symptoms were documented in 22% of cases [40]. The reduction in the percentage is likely mitigated by the chronic use of PPIs for life, which is necessary after the procedure. The effects of long-term GERD on patient outcomes are still limited.

### 3.6. Comparison with Other Interventions

In recent years, multiple studies have compared POEM to alternative interventions, including pneumatic dilatation (PD) and LHM, which in the past represented the gold standard for the treatment of achalasia.

In a multicentric RCT conducted by Ponds et al., comparing PD and POEM and involving 66 patients for POEM and 64 patients for PD (single session), the clinical success at 2-year follow-up was significantly higher in the POEM cohort compared to the PD cohort’s (92% versus 54%, *p* < 0.001) [44]. A recent study has confirmed the higher clinical success of POEM compared to PD even at a 5-year follow-up (81% versus 40%, *p* < 0.0001). However, in 5-year follow-up endoscopies, the reflux esophagitis rate was higher in the POEM cohort compared to the PD cohort (33% versus 13%), with no instances of severe esophagitis (Los Angeles grades C and D) observed after PD [45].

Werner et al. compared LHM with fundoplication and POEM in an RCT, which enrolled 221 treatment-naïve achalasia (16.2% type I, 73.7% type II, and 9.5% type III). The authors found that the 2-year clinical success rates were similar between surgical and endoscopic myotomies (83% for POEM and 81.7% for LHM). However, procedural adverse events, including mucosa perforations and hiatal stenosis, were more frequent after surgery compared to POEM (7.3% versus 2.3%). As expected, higher rates of reflux esophagitis were observed in the POEM cohort (44% versus 29% at 2 years). Nevertheless, the incidence of severe reflux esophagitis (Los Angeles grades C and D) appeared comparable 2 years after POEM and LHM (5% versus 6%), suggesting a potential diminishing efficacy of the fundoplication over time [46].

**Table 1:** Studies assessing the long-term outcomes of esophageal POEM in achalasia.

Author (Year)	Study Design	Number of Patients	Previous Treatment (%)	Myotomy Length (cm)	Definition of Clinical Success	Clinical Success	Predictors of Clinical Success	Adverse Events	Reflux Symptom *,#	Erosive Esophagitis #	Ref
Inoue et al. (2015)	Retrospective	500	39	17	ES ≤ 3	88.5% at 36 months	NA	3.2% of patients	19.4% at 1–2-year follow-up 21.3% at 3-year follow-up	59.2% at 1–2- year follow-up 56.3% at 3-year follow-up	[25]
Li et al. (2018)	Prospective	564	34.2	10	ES ≤ 3	94.2%, 92.2%, 91.1%, 88.6%, and 87.1% at 1, 2, 3, 4, and 5 years	NA	6.4% major adverse events	36.5%	17%	[26]
Zhang et al. (2023)	Metanalysis	2698	NA	NA	ES ≤ 3	91.3%, 90.4%, 89.8%, and 82.2% at 2, 3, 4, and 5 years	NA	NA	23.9%	16.7%	[27]
Zhang et al. (2020)	Prospective	32	NA	12	ES ≤ 3	88% at 88 months	NA	22% of patients	38%	9.3%	[28]
Modayil et al. (2021)	Prospective	610	47.9	10	ES ≤ 3	98%, 96%, 96%, 94%, 92%, 91%, and 91% at years 1, 2, 3, 4, 5, 6, and 7	NA	10.5% mucosotomies 3.4% of clinically significant adverse events	20.5% (pH study positive in 57.1% at median of 4 months)	48.8%	[29]
Kuipers et al. (2019)	RCT	64	0	NA	ES ≤ 3	81% at 60 months	Age < 40 years	No serious adverse events	NA	33.3% at 5 year-follow-up	[45]
Werner et al. (2019)	RCT	112	34.8	12.5	ES ≤ 3	83% at 24 months	NA	2.7% of patients (serious adverse events)	45.8%	44% at 24 months	[46]

RCT Randomized Controlled Trial GERD Gastroesophageal Reflux Disease ES Eckardt score. * Different reflux symptoms assessment methods were used among the studies; # follow-up data are not available for every patient.

### 3.7. POEM for Naïve Patients and after Failed Treatment

POEM has proven effective in treatment-naïve patients as well as in patients who have already undergone interventions [47,48]. Prior PD does not hinder POEMs technical and clinical success, although it may increase the degree of submucosal fibrosis [47]. A comprehensive meta-analysis revealed consistent clinical success (90%) alongside notable symptom and manometric improvement after POEM in patients previously unresponsive to LHM [41]. The potential for POEM to modify myotomy orientation (posterior) contributes to the efficacy of redo-treatment following surgical failure. A recent RCT comparing POEM and PD after failed LHM highlighted a 3-fold higher success rate of POEM (62.2% versus 26.7%, respectively), with comparable short-term reflux esophagitis rates [49].

### 3.8. Future Directions: Towards a Short, Tailored Myotomy

The initial POEM description by Inoue implied a 10 cm myotomy (7 cm esophageal, 3 cm gastric) [7]. However, considering that the LES is the crucial site of obstruction and manometric findings indicate a normal length of around 3 cm [50], this somewhat arbitrary extension contradicts physiological considerations. For all achalasia subtypes except type III, a shorter myotomy tailored to the LES is in line with the rationale of effectively relieving the obstruction while avoiding unnecessary submucosal tunneling and myotomy. This approach could potentially reduce the risk of intraprocedural adverse events, such as bleeding or perforation.

Additionally, preserving the gastric oblique non-spastic sling fibers crucial for LES competence by orienting the myotomy either anteriorly (1–2 o’clock) or posteriorly (4–5 o’clock) may also help reduce the risk of GERD [51,52].

The future directions of precision endoscopy in esophageal POEM management encompass this principle: validating a targeted myotomy as minimally extensive as possible while maintaining high clinical success rates and reducing the incidence of intra-procedural events and GERD.

Recently, our group reported the experience of performing POEM using a pediatric endoscope with the aim of reducing the extent of the submucosal tunnel and myotomy while achieving technical and clinical success (1-month follow-up) [53]. The disadvantage of using a slim endoscope lies in its inability to manage potential adverse events, such as bleeding, as the treatment devices do not fit through the small operating channel. The use of a smaller endoscope during POEM, along with the development of suitable devices, merits further consideration.

Emerging evidence of short myotomies is very promising.

In a first RCT, Gu et al. compared short myotomies (mean 5.6 cm) with standard myotomies (10 cm) in patients with type II achalasia. They demonstrated comparable treatment success (96% versus 94%), a shorter procedure time (31 min versus 46 min, *p* = 0.05), and reduced postoperative abnormal acid exposure on 24 h pH-metry (23.9% versus 43.8%, *p* = 0.042) in the short myotomy group [54].

In the RCT conducted by Nabi et al., 71 patients with type I and II achalasia were randomized to receive a very short myotomy (3 cm) or a long myotomy (6 cm and above). Clinical success after one-year follow-up was comparable between the two groups (93.55% short myotomy, 96.97% long myotomy, *p* = 1.0). Additionally, esophagitis rates and 24 h pH impedance study results showed no significant differences (esophagitis: 32.4% short myotomy, 48.7% long myotomy, and 24 h pH impedance: 40% long myotomy and 25.9% short myotomy, *p* = 0.399) [55].

In a recent RCT, Familiari et al. randomized 200 patients with achalasia into long POEM (13 cm) or short POEM (8 cm) arms. The authors revealed that shorter POEM was not inferior to long POEM in terms of clinical success rates at 24 months (98% versus 89.1%). They also found a reduced procedure time in the short POEM arm and observed no significant differences in GERD rates between the two groups at both the 6- and 24-month follow-up periods (pH-metry resulted as pathological in 34.3% in the long myotomy group versus 31.1% in the short myotomy group, while endoscopic GERD was reported in 37.6% versus 51.5% at 6 months and in 21% versus 24.5% at 24 months, respectively) [56].

It is worth highlighting the difference in the definition of “short” POEM among recent studies (Familiari et al. defined short as an 8 cm myotomy, consistent with the original description [7]), warranting future research to standardize the optimal short-POEM extension.

Although the data are promising, the adoption of short myotomies mainly scares endoscopists, primarily due to concerns about the possibility of an inadequate LES myotomy.

FLIP is an endoscopically used device that provides a real-time evaluation of esophageal physiology. Its use has recently been proposed to confidently identify the LES during POEM to guide tailored therapy. The most accurate intraoperative indicator for detecting obstruction is the esophagogastric junction distensibility index (EGJ-DI) at 60 mL fill [57,58].

Recent evidence has assessed the use of FLIP for guiding endoscopic myotomies. In the study conducted by Holmstrom et al., the group of patients treated with FLIP-guided myotomy showed significantly higher clinical success rates after 12 months compared to the non-FLIP cohort (93% versus 81%, *p* < 0.05). Notably, intraoperative FLIP use led to additional myotomies in over half of the cases [59]. In a British prospective study, a FLIP-guided short POEM (less than 7 cm) exhibited a 6-week symptom resolution rate of 93%, along with lower postoperative proton-pump inhibitor (PPI) use (40%) when compared to longer myotomies (60%) [60]. As extended myotomies may weaken the esophageal wall, potentially increasing the risk of Blown-out myotomies (BOM), and may not effectively relieve functional obstruction, there has been a proposal for lateral extensions of myotomies guided by intraoperative FLIP in cases where LES myotomies appear inadequate [61]. Randomized trials of FLIP-guided short myotomies are currently ongoing and will help determine whether this approach may represent the future direction of esophageal POEM [62].

## 4. Gastric Peroral Endoscopic Myotomy (GPOEM)

GP is a chronic condition characterized by delayed gastric emptying (GE) in the absence of mechanical obstruction.

The primary symptoms, which typically recur, include nausea, vomiting, early satiety, postprandial fullness, stomach distension, and bloating [63]. The disease can become highly debilitating and significantly impair the quality of life [64]. The Gastroparesis Cardinal Symptom Index (GCSI) score is a validated questionnaire based on the evaluation of three cardinal groups of symptoms (nausea and vomiting, fullness and early satiety, bloating, and distension) used to assess the severity of the disease [65,66].

The gastric emptying study (GES) is the gold standard for GP diagnosis, although other diagnostic techniques have been recently validated for clinical practice, including wireless motility capsules (WMCs) and the 13C-breath test for GE [67,68].

GP pathogenesis is not yet well defined. Multiple mechanisms contribute to the complex pathophysiology of the disorder, including vagal nerve dysfunction, visceral hypersensivity, impaired fundic accommodation (FA), antral hypomotility, gastric dysrhythmias, and increased pylorus tone, defined as “pylorospasm” [68]. It has been speculated that the microbiota and duodenal dysbiosis influence the development of GP [69], as seen in other gastrointestinal diseases [70,71,72,73].

Various etiologies are associated with GP, including diabetes [74], surgical interventions [75], myopathic disorders [76,77], neurological conditions [78], and bacterial and viral infections [69]. However, in the majority of cases, an etiology is not identified, and GP is defined as idiopathic [69].

Medications and dietary management are first-line therapies for GPs, but approximately one-third of patients do not benefit from conservative treatments [79]. GP is considered refractory if there is a poor response to a 6-month treatment with dietary modifications and a trial of the maximum tolerated dose of prokinetic therapy [80].

The theory that pylorospasm may be involved in the pathogenesis of GP has led to the development of multiple pylorus-related therapies for refractory GP. Over the years, several endoscopic and surgical techniques have been developed, including botulinum toxin injection (TBI), trans-pyloric stent placement, and laparoscopic pyloromyotomy. However, these techniques have been associated with limited efficacy (TBI) [81], high rates of adverse events (stent migration) [82], or suboptimal long-term effectiveness (surgical pyloromyotomy) [83].

Initially reported in 2012 with an initial application in a porcine model [84] and the first human performance in 2013 by Kashab et al. [85], Gastric Per-Oral Endoscopic Myotomy (GPOEM) has emerged as a non-invasive approach to pyloromyotomy, extending the technique that has revolutionized achalasia therapy.

Over the last ten years, G-POEM has been associated with encouraging efficacy data and low rates of adverse events. The latest European Society of Gastrointestinal Endoscopy (ESGE) guidelines recommend considering G-POEM in patients with refractory GP in expert centers and suggest caution against other endoscopic treatments [86]. Although long-term data are currently lacking, GPOEM is a viable option for GP.

### 4.1. Technique

G-POEM applies the principles of third-space endoscopy, mirroring the procedural steps of E-POEM.

**Figure 2:**
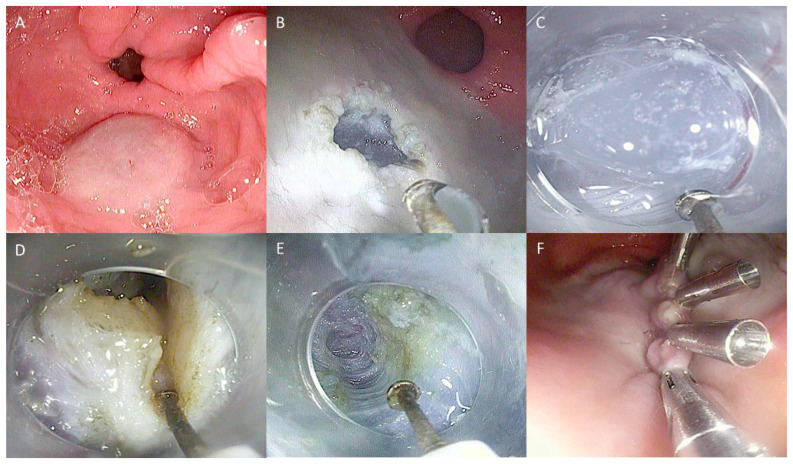
Technical steps of G-POEM: (**A**) Submucosal injection; (**B**) Mucosal incision to create tunnel entrance; (**C**) Submucosal tunnelization; (**D**) Pyloromyotomy (**E**) View of the tunnel at the end of myotomy; (**F**) Closure of the mucosal incision with endoclips. The copyrights to the pictures belong to the authors.

After creating a submucosal cushion by injecting a solution mixed with saline, epinephrine, and indigo carmine, a mucosal incision is made 5 cm from the pylorus on the greater curvature of the stomach. A submucosal tunnel is then created, extending approximately 1 cm beyond the pylorus. The myotomy is performed from 2–3 cm proximal to the pylorus and extends 1 cm beyond the duodenal bulb. The initial mucosal entry point is either closed or sutured (Figure 2) [87].

G-POEM poses greater technical challenges compared to E-POEM due to issues such as scope looping in the stomach, a higher risk of major bleeding from the gastroduodenal artery territory, potential duodenal perforation, tunnel curving over the pyloric muscle, scope instability due to gastric contractions, and less distinct anatomical landmarks [88].

While the access point is typically chosen on the greater curvature of the stomach, some operators have reported using mucosal entries on the lesser curvature, posterior wall, or anterior wall [89]. Mucosotomy is generally performed longitudinally and closed using TTS clips or endoscopic suturing systems [90]. In a prospective study conducted by Hustak et al., involving 40 patients, no different efficacy rates in mucosal closure were found when comparing TTS-clips and the OverStitch system (Apollo Endosurgery, Austin, TX, USA) and through-TTS (89% versus 100%, *p* = 0.49) [91].

The dissection of the tunnel follows similar current settings as applied in E-POEM. Nevertheless, unlike POEM, it is recommended that myotomy be performed using “safe” knives (e.g., the IT-2 knife, Olympus, Westborough, MA, USA) to mitigate the risk of duodenal perforation [88].

Technical variations of G-POEM include different orientations of the myotomy, partial versus full-thickness myotomy, and single versus double myotomy. While a single study reported a higher clinical success rate in the double myotomy compared to the single myotomy, further data are still necessary to determine the optimal approach [90].

### 4.2. Outcomes

The efficacy of G-POEM is defined as clinical improvement, as measured recently by a one-point decrease in the GCSI score with a ≥25% decrease in two subscales [68].

Table 2 provides an overview of primary studies assessing the outcomes of G-POEM in patients with refractory GP.

In the first multicenter prospective study involving 30 patients, G-POEM achieved a clinical success rate at the six-month follow-up of 86% [92]. A pooled analysis by Spadaccini et al., involving 10 studies and 292 patients, found that G-POEM led to symptomatic improvement in 83.9% of cases after a mean follow-up of 7.8 months [89].

In 2021, a study led by Vosoughi and Kashab, conducted across 5 US centers with 80 GP patients, consistently assessed the long-term effects of G-POEM for the first time. Clinical success, defined as at least a one-point decrease in GCSI score with a ≥25% reduction in two subscales, was 56% at 12 months. A baseline GCSI Score > 2.6 (OR 3.23, *p* = 0.04), gastric retention > 20% at 4 h (OR 3.65, *p* = 0.03), and an early response to G-POEM (1 month after treatment) (OR 8.75, *p* < 0.001) were independent predictors of clinical success [93].

Subsequently, a multicenter French study involving 76 patients treated with G-POEM reported a 1-year clinical success of 65.8%, with a median reduction rate in the overall GCSI score of 41%. Notably, a high preoperative GCSI satiety subscale score predicted clinical success (OR 3.41, *p* = 0.048), while a high rate of gastric retention at 4 h correlated with clinical failure (OR 0.97, *p*= 0.03) [94].

In the study by Abdelfatah et al., involving 90 patients, the clinical success of G-POEM was 73% at 6 months, 65% at 1 year, 51% at 2 years, and 45% at 3 years. A high BMI (OR 1.097, *p*= 010) and a history of psychiatric medications (OR 1.33, *p* = 0.052) were found to be associated with the risk of G-POEM failure [80].

The meta-analysis conducted by Kamal et al., including 10 studies assessing the 1-year outcome of G-POEM until June 2021 (a total of 482 patients), showed a pooled clinical success of 61% and a significant reduction in the mean post-procedural GCSI compared to the pre-procedural score, with a mean difference of −1.4 [95].

In the multicentric study conducted by Labonde et al., which enrolled 65 patients, the clinical success of G-POEM was 65.2% at 36 months. In a scoring system that included nausea GCSI subscale < 2 (1 point), satiety GCSI subscale > 4 (1 point), bloating GCSI subscale > 3.5 (1 point), and gastric retention at 4 h > 50% (1 point), a threshold of 2 was found to predict clinical success with a sensitivity of 93.3% and a specificity of 56.3% [96].

A recent large prospective multicenter study involving 374 GP patients (including 141 with diabetic GP) revealed a 4-year clinical success rate of 77.5% (79/102 patients), with diabetic etiology having the most favorable outcome (86% clinical success), compared to idiopathic (72.5% clinical success, *p* = 0.003) and post-surgical GP (68.8% clinical success, *p* < 0.001). Notably, diabetic etiology was also associated with a more prominent improvement in GES findings after myotomy. However, GES normalization was relatively consistent across etiologies and did not significantly correlate with changes in clinical symptoms [97].

While suboptimal percentages of clinical success and the lack of correlation with GES findings have raised reasonable concerns about the therapeutic value of G-POEM, the first sham-controlled RCT, comparing G-POEM outcomes with a placebo (upper endoscopic examination without pyloromyotomy), demonstrated the unbiased utility of the procedure in the treatment of refractory GP. The clinical success rate, defined as a decrease of at least 50% in GCSI, was 71% after G-POEM, compared to 22% in the sham group (*p* = 0.005). Interestingly, the efficacy rate was found to be higher in diabetic GP compared to idiopathic and postsurgical GP (89% versus 67% and 50%, respectively). Notably, G-POEM resulted in an overall improvement in GES findings (median gastric retention at 4 h was reduced from 22% to 12% after G-POEM) and increased pyloric distensibility [98].

G-POEM is an effective strategy for GPs following surgical interventions. In the study by Tan et al., which enrolled 79 GP patients who had undergone proximal gastrectomy and esophagectomy, a clinical response, defined as a more than 25% decrease in at least two subscales of the GCSI score, was achieved in 78.3% of patients at 12 months and in 81.8% (27 out of 33) of patients at 24 months [99]. A recent meta-analysis of 4 observational studies (104 patients), assessing the outcomes of G-POEM as a treatment for post-lung transplant GP, reported a pooled estimated clinical efficacy of 83%, with a mean reduction in GCSI score following the procedure of −2.01 [100].

### 4.3. Adverse Events

G-POEM is generally considered a safe procedure. The most common intraprocedural or short-term adverse events include abdominal pain, capnomediastinum/capnoperitoneum, antral/prepyloric ulceration, bleeding, and mucosal injury. In the meta-analysis by Kamal et al., the pooled rate of adverse events was 8% [95]. A similar rate (8.6%) was reported in the study by Hernandez Mondragon et al., which had a larger case series following the meta-analysis [97].

Most complications after G-POEM are mild and self-limiting (such as abdominal pain and capnoperitoneum) or can be managed endoscopically (bleeding). However, perforation, although potentially treatable endoscopically (with a stent or clip), might also require surgical intervention and generally appears to carry a more challenging outcome compared to E-POEM [88]. The long-term adverse effects of G-POEM are not currently known, and further data will be necessary to define them.

**Table 2:** Studies assessing the outcomes of G-POEM.

Author (Year)	Study Design	Number of Patients	Etiology	Definition of Clinical Success	Clinical Responders	Predictors of Clinical Success	Predictors of Clinical Failure	Adverse Events	GES Changes	Ref
Khasbah et al. (2017)	Retrospective	30	11 diabetes 7 idiopathic 12 postsurgical	Improvement in GP symptoms with the absence of recurrent hospitalization	86% at 6 months	NA	NA	6.7% of patients	Improvement in 82% of patients, normalization in 47% of patients	[92]
Vosoughi et al. (2021)	Prospective	75	18 diabetes 31 idiopathic 26 postsurgical	One point decrease in the GCSI score with a ≥25% decrease in two subscales	56% at 12 months	GCSI score > 2.6, gastric RI > 20% at 4 h, and early response at 1 month		6% of patients	Normalization in 47% of patients	[93]
Ragi et al. (2020)	Retrospective	76	26 diabetes 27 idiopathic 23 postsurgical	At least a 1-point improvement in the GCSI score	66% at 12 months	High GCSI satiety subscale	High rate of RI at 4 h	6% of patients	Decrease in 2 h and 4 h RI (65 patients assessed)	[94]
Abdelfatah et al. (2021)	Retrospective	97	38 diabetes 42 idiopathic 10 others	One point decrease in the GCSI score with a ≥25% decrease in two subscales	75% at 6 months 69% at 12 months	NA	High BMIs and history of psychiatric medications	4% of patients	Improvement in 90% of patients Normalization in 63% of patients (74 patients assessed)	[80]
Labonde et al. (2022)	Prospective	46	15 diabetes 16 idiopathic 9 postsurgical 6 others	One point decrease in GCSI	65% at 36 months	Lower GCSI nausea subscale, higher satiety, and bloating	Higher GCSI nausea subscale	NA	NA	[96]
Hernandez Mondragon et al. (2022)	Retrospective	374	141 diabetes 115 idiopathic 102 postsurgical 16 others	One point decrease in the GCSI score with a ≥25% decrease in two subscales	77.5 at 48 months	Diabetic etiology, early diagnosis of gastroparesis < 24 months, predominant nausea and vomiting, GCSI score 1.5–2.5 at 6 months, RI at 4 h < 10% at 6 months	NA	8.6% of patients	Normalization in 63.9% of patients at 48 months	[97]
Martinek et al. (2022)	RCT	41	17 diabetes 11 idiopathic 13 postsurgical	Improvement in GCSI by at least 50%	71% at 6 months	Male gender, RI at 4 h > 20% and post G-POEM pyloric distensibility ˃ 13 mm^2^/mm Hg at 40 mL	NA	9% of patients	Decrease in median gastric retention at 4 h (from 22% to 12%)	[98]

GCSI Gastroparesis Cardinal Symptom Index, NA Not available, RCT Randomized Controlled Trial, GP gastroparesis, RI Retention Index.

### 4.4. Comparison with Other Interventional Treatments

G-POEM has demonstrated superiority over other therapeutic approaches for refractory GP. In the retrospective study conducted by Landreneau et al., comparing G-POEM and surgical pyloroplasty, the two techniques resulted in similar yet significant improvements in GCSI scores and GE. However, the surgical pyloroplasty cohort reported more complications (16.7% versus 3.3%, *p* = 0.086), including surgical site infections (6.7 versus 0%), pneumonia (6.7 versus 0.0%), and ICU admissions (10.0 versus 0.0%). Notably, patients who underwent pyloroplasty had a longer average length of stay, operative time, and estimated blood loss [101]. In the multicenter study conducted by Pioppo et al., G-POEM demonstrated a higher reduction in post-operative GCSI scores (*p* < 0.001) when compared to surgery and a reduction in post-operative retention at both 2 and 4 h as well. Furthermore, endoscopic pyloromyotomy resulted in shorter hospital stays, lower rates of adverse events, and lower mean blood loss compared to pyloroplasty [102].

G-POEM has shown superiority over gastric electrical stimulation, which until recently was considered the only non-surgical option for refractory GP. In a retrospective propensity score-matched analysis that compared the two treatments (23 patients per treatment group, median follow-up of 27.7 months), G-POEM exhibited higher clinical success (76.6% versus 53.7% at 24 months) and a lower rate of adverse events (26.1% versus 4.3%). Interestingly, the clinical efficacy of G-POEM was better in idiopathic GP [103].

### 4.5. Future Directions: Selecting Patients to Improve Outcomes

G-POEM stands as a promising and safe technique for refractory GP; however, it faces enduring and substantial challenges. The RCT conducted by Martinek et al. has effectively addressed doubts about the efficacy of G-POEM in treating refractory GP and has demonstrated its superiority over a sham procedure [98]. Nevertheless, the observed suboptimal success and the diminishing post-procedural efficacy in long-term studies suggest the presence of intricate factors within the realm of GP management that require a more comprehensive understanding.

Recent research has unveiled how multiple dysfunctions can contribute to the clinical manifestation of GP, extending beyond pylorospasm. These include visceral hypersensitivity, impaired FA, and reduced antral contractions [104,105]. This prompts the question of whether addressing the pylorus can truly resolve the underlying organic dysfunction in all cases, considering the intricate nature of the disorder.

While GES remains the gold standard diagnostic method for GP, it is becoming increasingly evident that delayed GE in refractory disease cannot be the sole criterion for indicating endoscopic myotomy [106,107,108,109].

The future advancement of G-POEM to enhance outcomes lies in delving into and identifying the individualized pathophysiological mechanisms of GP. This is the purpose of precision treatment of the disease, aiming to identify patients who could benefit from pylorus-targeted treatment.

Although GES might be considered to provide limited information, this method can offer intriguing insights into the regional distribution of food within the stomach, potentially shedding light on the physiopathological mechanisms involved in the disease. In the study conducted by Mandarino et al., which involved 21 patients undergoing G-POEM, a lower median pre-procedural IMD0 value, defined as the ratio of gastric counts in the proximal stomach to the total stomach at time 0 at pre-procedural GES, was associated with higher rates of functional response (a decrease of more than 30% in 2 h retention in GES) after G-POEM. A lower IMD0 value indicates antral food retention, likely linked to impaired FA. This suggests that patients with more distally located gastric disease may benefit the most from endoscopic pyloromyotomy [110].

New diagnostic techniques beyond GES will need to be employed to identify GP patients eligible for G-POEM. FLIP, the innovative method that has revolutionized GI sphincter evaluation, has the potential to identify patients who could benefit from pylorus-targeted therapy by quantifying pyloric hypertonicity. In the study conducted by Jacques et al., a low pre-therapeutic Pylorus Distensibility Index (P-DI) value (<9.2 mm^2^/mmHg) was found to predict clinical success at 3 months after G-POEM, with a sensitivity of 100% and a specificity of 72% [111]. Further research is needed to establish the recommendation of the technique as a screening procedure.

Novel diagnostic tools such as high-resolution electrogastrography have shown great promise in advancing our understanding of the physiology of gastric dysmotility [112,113], aiding in the evaluation of individual patients, and guiding the selection of targeted therapies.

Precision treatment in GP could also involve the analysis of cellular alterations that have been shown to be associated with GP in order to characterize the disease. Changes related to GP include the loss of interstitial Cajal cells (ICCs), alterations in the enteric nervous system, and smooth muscle cells, as well as dysregulation in the macrophage population, specifically a reduction in the number of anti-inflammatory macrophages [114]. However, according to the most recent guidelines by the American Gastroenterology Association, full-thickness biopsies should currently primarily be reserved for research purposes [115]. So far, only one study conducted by Shah et al. has included muscular biopsies during G-POEM. Interestingly, a higher average number of ICCs in gastric biopsies was found to be correlated with clinical success after G-POEM [116]. The role of full-thickness biopsies for ICC count during G-POEM requires further extensive clarification. However, this could also potentially be part of the future personalized management of patients with GPs, aimed at selecting ideal candidates for pyloromyotomy.

## 5. Zenker Diverticulum

ZD, the most common type of esophageal diverticulum, is a protrusion of the mucosal and submucosal layers through the muscle fibers of the Killian triangle. This area of relative weakness is located in the posterior wall of the pharynx and is bordered laterally by the thyropharyngeal and superiorly by the cricopharyngeal fibers of the superior constrictor pharyngeal muscle [117].

The exact etiopathogenesis of ZD remains unclear. However, the primary contributing factor has been identified as the incoordination of muscular contractions during swallowing, resulting in consistent pressure on the fixed sphincter. This leads to an outpouching of the hypopharyngeal esophageal wall through a region known as “locus minoris resistentiae” [118].

Common symptoms of ZD include halitosis, dysphagia, regurgitation, and chest pain. Nevertheless, most patients remain asymptomatic [119,120]. According to ESGE guidelines, treatment is recommended upon the onset of symptoms, with a comprehensive assessment of the risk-benefit ratio, regardless of the size of the diverticulum [121].

Historically, rigid endoscope diverticulectomy and surgical procedures involving myotomy of the upper sphincter and pouch resection were considered the gold standard for treatment. In the 1990s, the first endoscopic technique introduced was flexible endoscopic septotomy diverticulectomy (FESD) [122,123,124]. This procedure involves a thorough full-thickness incision of the multi-layered septum, similarly to the surgical approach [125], and it is still employed in numerous centers today. In a meta-analysis of 20 studies, FESD demonstrated high rates of clinical success (92%), a low recurrence rate (11.6%), and a somewhat sub-optimal safety profile (overall adverse events rate of 11.3%) [126].

In recent years, advancements in interventional endoscopy have given rise to novel techniques in the therapeutic arsenal for ZD, significantly transforming the management of symptomatic patients (Table 3).

### 5.1. Zenker-Peroral Endoscopic Myotomy (Z-POEM)

#### 5.1.1. Technique

Z-POEM was initially introduced by Li et al. in 2016 [127] and subsequently termed Z-POEM by Hernàndez Mondragòn and colleagues [128]. The technique was developed as an adaptation of the principles of POEM for the treatment of ZD.

The procedure commences with the creation of a submucosal bleb approximately 3 cm proximal to the diverticular septum using a saline mixture like that used in POEM. A 1.5–2 cm longitudinal mucosal incision is made at the intended tunnel entry site. Then, tunneling is undertaken on both sides of the septum. The commonly employed knife is the Hybrid knife (ERBE USA Inc., Marietta, GA, USA) [129]. The tunnel is extended caudally on each side, terminating 1–2 cm below the base of the diverticulum. Following this, the myotomy of the cricopharyngeal septum is performed. The final step involves meticulous hemostasis and closure of the mucosal incision site using TTS-clips. The main challenge of Z-POEM lies in its technical complexity, starting from the initial approach to the challenging anatomical location and extending to the effective closure of the mucosal defect. The reported high rates of technical success are likely influenced by the fact that these studies on Z-POEM are conducted in highly specialized centers [88].

#### 5.1.2. Outcomes and Comparisons with Other Techniques

The assessment of symptoms in ZD varies across studies and utilizes several scoring systems, including the Eckardt score [22], the Dakkak Bennett dysphagia numerical score [130], as well as the Kothari-Haber score [131,132]. There is no standardized definition of clinical success after Z-POEM. The most commonly used criteria in the literature include a broad decrease in the Dakkak Bennett score (to 0–1) or an Eckardt score drop of <3, along with a complete resolution of dysphagia.

Since the initial reports, Z-POEM has demonstrated a high level of 12-month clinical efficacy, ranging between 86% and 92% [133,134]. An early meta-analysis of 10 studies up to 2020 exhibited a clinical success of 97.1%, defined as the complete resolution of symptoms at 1-year follow-up [135]. In the meta-analysis conducted by Facciorusso et al., involving 12 studies (300 patients), the pooled clinical success rate after Z-POEM (defined as the decrease of Eckardt score < 3 in 3 studies, Dakkak Bennett score reduction to 0 or 1 in 5 studies, improvement of dysphagia in 1 study, not specified in 2 series) was 94% [136]. The most recent systematic review with a meta-analysis of available Z-POEM studies reported an overall pooled clinical efficacy of 93.0% [137]. 

Recently, Stainway and colleagues conducted an international multicenter study involving 89 patients to evaluate the long-term efficacy of Z-POEM. They reported an average improvement in all components of the Dakkak Bennett score after a mean follow-up of 37 months (range 24–63 months): dysphagia (preprocedural 2.1—postprocedural 0.13, *p* < 0.001), regurgitation (preprocedural 2.8—postprocedural 0.11, *p* < 0.001), and respiratory scores (preprocedural 1.8—postprocedural 0.05, *p* < 0.0001). Symptom recurrence was observed in 6.7% of patients [138].

The pooled technical success of the procedure was consistently reported as excellent, with rates ranging from 95% to 97% [135,136,137].

Recurrence rates after Z-POEM vary among studies, ranging from 0% to 11.2% [136,139]. To date, no consistent predictive factors (such as symptom severity, diverticulum size, or prior treatments) have been associated with symptom recurrence [137,138].

Several studies have compared Z-POEM with the more traditional FESD. In an early study by Al Ghamdi et al., enrolling 245 patients and comparing Z-POEM, FESD, and rigid diverticulotomy, clinical success rates were similar among techniques (92.7%, 86.7%, and 89.2%, *p* = 0.26). No statistical differences in technical success (95%, 95%, and 87.5%, respectively, *p*= 0.18) and recurrence rates (15 Z-POEM, 6 FESD, 3 rigid, *p* = 0.47) were reported, and Z-POEM had a longer procedural time compared to FESD (46.13 min versus 33.72 min). Adverse events occurred in 30.0% rigid septotomy patients, 16.8% ZPOEM patients, and 2.3% flexible septotomy patients (*p* < 0.05) [139].

In a recent study by Swei et al., Z-POEM exhibited superior long-term efficacy compared to FESD (100% versus 86.7%, respectively, *p*= 0.18), and comparable technical success (100% for both), while none of these outcomes reached statistical significance (probably due to the small sample size). Safety was comparable between the two techniques, with adverse event rates of 0% and 3.9%, respectively [140].

Currently, no relevant data exists comparing the outcomes of the technique between treatment-naïve and previously treated patients. Only one study, conducted by Sanaei et al. and enrolling 32 patients, has assessed the outcomes of Z-POEM in previously endoscopically or surgically treated patients. This study reported 93.8% technical success with a clinical success rate of 96.7%. The authors concluded that Z-POEM is feasible and effective in most patients with recurrent symptoms after previous treatment [141].

#### 5.1.3. Adverse Events

Z-POEM is considered a safe procedure, but it should be conducted with careful attention. Intraprocedural complications reported include emphysema, pneumoperitoneum, perforation, aspiration pneumonia, fever, and bleeding. A systematic review and meta-analysis of nine studies comprising 195 patients estimated an intraprocedural adverse event rate of 2%. Emphysema linked to the passage of CO2 during the procedure, as well as potential perforation, was the most common effect. In a subgroup analysis, the intra-procedural perforation rate was 6% [141]. In the meta-analysis conducted by Facciorusso et al., the overall pooled adverse event rate after Z-POEM was 10.6%, with 3.5% of serious adverse events [136]. Another meta-analysis reported a pooled adverse event rate of 6% [142].

### 5.2. Alternative Third-Space Techniques

In recent years, new techniques have been developed to overcome the technical challenges associated with Z-POEM.

#### 5.2.1. Peroral Endoscopic Septotomy (POES)

As anatomical constraints and pharyngeal muscular contractions may hinder the optimal creation of a proximal entry to the diverticulum septum, Repici et al. proposed an alternative submucosal approach involving direct mucosal incision and submucosal tunneling at the top of the diverticulum in a lengthwise direction. This technique was termed Peroral Endoscopic Septotomy (POES) [143]. In their initial experience, the authors revealed a clinical success, defined as improvement of dysphagia, of 95% (20/21 patients) [143]. Other approaches, technically similar, have been proposed by Hu et al. and Klingler et al., reporting suboptimal clinical outcomes [144,145].

The primary advantage of POES lies in its significantly reduced procedural time, with a mean of 13.8 min [143]. However, elevated rates of adverse events (up to 21%) warrant a cautious approach [145,146].

#### 5.2.2. Hybrid Techniques

**Figure 3:**
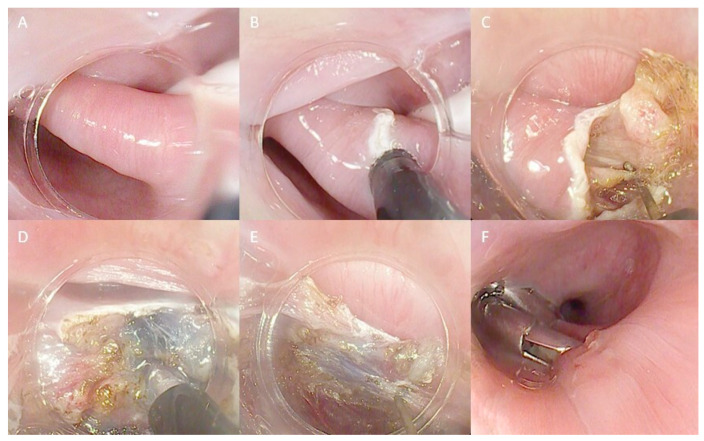
Technical steps of the hybrid technique myotomy for ZD treatment: (**A**) ZD view with a nasojejunal tube in the esophageal lumen used as a landmark; (**B**) First incision of the septum; (**C**) Initial septotomy with a freehand technique; (**D**) Submucosal injection to better distinguish submucosal and muscular layers; (**E**) Continuing the septotomy, repeating injection when necessary; (**F**) Final closure with endoclips. The copyrights to the pictures belong to the authors.

Hybrid techniques combine principles from classical FES with those involved in submucosal dissection (Figure 3). An example of this is Peroral Endoscopic Diverticulotomy (POED), as described by Pugliese and colleagues. The technique utilizes a soft diverticuloscope and involves a myotomy of the cricopharyngeal muscle from proximal to distal, performing the diverticulotomy up to the last centimeter from the diverticular base. At this stage, submucosal injection is performed on both the diverticular and esophageal sides to enhance the exposure of the muscular fibers (147). Resection margins extend onto the esophageal muscles beyond the diverticular base, and finally, endoclips are applied at the resection margins [147,148]. In our center, we perform a variant of the technique without utilizing the diverticuloscope (Figure 3).

Although limited by small case series, POED demonstrated high clinical and technical success rates (both 100%) and a short procedure duration (averaging 20 min, ranging from 12 to 30 min) [147,148].

### 5.3. Future Directions: Need for New Data for a Unique Endoscopic Myotomy Algorithm

The advancement of interventional endoscopy has enabled the development of techniques capable of treating ZD, reducing the necessity for surgery. As a result, patients now have access to less invasive and potentially more comfortable treatments.

However, further data are needed to refine the strategy for endoscopic ZD treatment.

FESD was the initial technique used and is known for its speed and effectiveness in surpassing open surgery, both in terms of practicality and the occurrence of adverse events. On the other hand, Z-POEM represents an evolution of the ZD resection techniques, incorporating third-space techniques into ZD. The advantages of third-space techniques include technical improvements, particularly in submucosal tunnelization, which provides enhanced access for a comprehensive myotomy of the entire septum. However, comparative studies between Z-POEM and FESD have not revealed better outcomes for Z-POEM or fewer adverse events or recurrences. Additionally, Z-POEM requires high expertise in third-space endoscopic approaches, often resulting in longer procedural times and higher costs. After Z-POEM for large ZD cases, the voluminous residual flap left after the intervention may lead to dysphagia recurrence [149]. In this specific scenario, considering additional flap mucosotomies is a precaution to avoid recurrences [150].

Further long-term prospective data are needed for Z-POEM to clearly establish the superiority of the third-space approach to ZD compared to traditional FESD.

Similar to E-POEM, tailoring the myotomy length for ZD to achieve symptomatic resolution without unreasonably increasing adverse events is still an issue to be addressed [151]. New evidence aimed at standardizing key technical aspects such as mucosal incision location and size, tunnel creation methods, and closure techniques could help optimize outcomes and safety profiles.

To address the technical challenges presented by the Z-POEM approach, techniques like POES and hybrid approaches such as POED have emerged as viable compromises between the benefits of the submucosal approach and the practicality of FESD. However, POES still requires longer follow-up data from robust, perspective-randomized studies, and POED necessitates studies with larger sample sizes.

The precision endoscopic treatment of ZD calls for new comparative evidence among endoscopic myotomies, ideally based on clinical trials, to establish the superiority of one technique over another or to develop a treatment algorithm that incorporates all available methods. In this context, validated patient-reported tools are essential for assessing symptoms and outcomes. Over the years, non-disease-specific scoring systems like the Eckardt score have been used to evaluate treatment outcomes for ZD. However, these scoring systems have not fully captured the specific range of symptoms associated with the disease. In this context, the Kothari-Haber score is emerging as a promising, validated tool designed to specifically address the distinctive features of ZD.

As the landscape of endoscopic interventions for ZD continues to evolve, there is an urgent need to establish a comprehensive body of evidence. This effort should encompass the evaluation of endoscopic techniques, their long-term durability, potential complications, and their applicability to patient and diverticulum characteristics. Such an approach would enable a tailored selection of techniques based on the patient’s profile, symptoms, and diverticulum size.

## 6. Third—Space Myotomies in Other Esophageal Conditions

### 6.1. Diverticular Peroral Endoscopic Myotomy (D-POEM)

Esophageal epiphrenic diverticulum (EED) is an outpouching from the distal esophagus that often co-occurs with motility disorders [152,153]. Surgery represents the primary therapeutic option, but it is linked to frequent complications, especially leaks (up to 20%) [154,155,156,157]. Additionally, progression to carcinoma arising within the diverticulum is a rare but documented occurrence [158].

Diverticular peroral endoscopic myotomy (D-POEM), following the principles of submucosal endoscopy, has developed as a technique for EED treatment.

The procedure involves making a vertical mucosal incision just above the diverticulum edge, parallel to the opening. The location is typically posterior but adapted to the diverticulum’s position. Submucosal tunneling is extended along the diverticular and septal submucosa, approximately 3 cm below the cardia. Subsequently, a septotomy is performed, followed by an antegrade myotomy up to 2 cm below the cardia. Finally, the mucosal defect is closed with TTS-clips [159]. In the initial multicentric experience conducted by Yang et al., the treatment success rate after D-POEM was 100%, with a technical success rate of 90.9% [160]. There were no adverse events. The recently available follow-up data at 2 years from the cohort of Nabi and colleagues showed a clinical success of 84.6% at 25 months after D-POEM [161].

Despite the promising data of D-POEM, some studies suggest that the treatment of symptomatic patients with EED could be effectively accomplished by lower esophageal sphincter myotomy [162,163]. A treatment algorithm based on diverticulum size and motility features is needed to define a tailored approach for EED [164]. As proposed by Samanta et al., septotomy should be considered for larger EEDs without motility disorders and avoided for small diverticula with non-relaxing sphincters [165].

### 6.2. Cricopharyngeal Peroral Endoscopic Myotomy (C-POEM)

It is an endoscopic technique for treating cricopharyngeal bar [166,167] or oropharyngeal dysphagia associated with Parkinson’s disease [168]. The procedure involves a submucosal injection and a mucosal incision performed approximately 2 cm above the cricopharyngeal muscle. Similar to POEM, a submucosal tunnel is created through mucosal access, followed by myotomy. The initial mucosal incision is closed with TTS-clips [166]. 

**Table 3:** Metanalysis assesses the outcomes of endoscopic treatment of esophageal diverticula.

Author (Year)	Study Design	Number of Patients	Techniques (*n*)	Procedural Time (min)	Technical Success	Clinical Success	Adverse Events	Follow-Up (Months)	Recurrence Rate	Ref
Ishaq et al. (2016)	Metanalysis	813	FESD (813)	NA	92%	88.0%	11.2% of patients	7–43	11%	[126]
Ren et al. (2022)	Metanalysis	153	Z-POEM (106) D-POEM (47)	NA	99%	94.1%	2% of patients	3–24	0	[132]
Spadaccini et al. (2021)	Metanalysis	196	Z-POEM (133) POES (63)	46.4 20.4	97.1% 96.5%	94.1% 91.1%	4.9% of patients	9–12	NA	[135]
Facciorusso et al. (2022)	Metanalysis	300	Z-POEM (260) D-POEM (40)	44.7 61.7	90.6% 87.7%	90.6% 94.2%	10.6% of patients 8.4% of patients	24	10.4%	[136]
Zhang et al. (2022)	Metanalysis	357	Z-POEM (357)	NA	96.3%	93.0%	12.4% of patients	24	11.2%	[137]
Kamal et al. (2021)	Metanalysis	233	Z-POEM (205) D-POEM (28)	NA	95% 97%	SMD 2.34 SMD 2.17	6.0% of patients 6.0% of patients	NA NA	NA NA	[142]
Mandavdhare et al. (2021)	Metanalysis	341	POEM (297) D-POEM (43)	NA	95.3% 94.4%	88.2% 91.4%	10.2% of patients 5.6% of patients	NA	NA	[165]

FESD Flexible Endoscopic Septum Division Z-POEM Zenker Peroral Endoscopic Myotomy D-POEM Diverticula Peroral Endoscopic Myotomy SMD Standardized Mean Difference.

The application of this technique is in its infancy. The first case series of C-POEM on three cases of cricopharyngeal bars has revealed dysphagia resolution in all patients [166]. Future studies with larger samples are warranted.

## 7. Conclusions

In recent years, POEMs have emerged as true game-changers in the landscape of UGT motility disorders. Esophageal POEM has been firmly established as the primary treatment for achalasia and other esophageal disorders, while G-POEM has demonstrated its remarkable efficacy in addressing refractory GP. Furthermore, several endoscopic myotomy techniques, including Z-POEM, have established themselves as safe and viable options for the treatment of ZD. Recently, a new concept of myotomies has been proposed for the treatment of EED and cricopharyngeal bar.

The advancement of interventional endoscopy has shifted the treatment choice away from surgery, not only in motility disorders but also in various other conditions, including complications after UGT tract surgery [169,170,171,172,173,174]. This paradigm shift reflects the increasing acceptance and trust in endoscopic approaches as effective and less invasive solutions.

While endoscopic myotomies are already widely integrated into clinical practice, they come with new challenges that need to be addressed. The concept of precision endoscopy in myotomies in UGT is poised to tailor myotomies, select the most appropriate patients, and unify myotomy techniques into a unique standardized treatment algorithm. This transformative concept aims to further enhance outcomes, reduce the incidence of adverse events, and reduce healthcare costs. The ongoing pursuit of new data and insights is essential to continually refine and optimize these treatments.

## Data Availability

Not applicable.
